# Beneficial effects of intravenous iron therapy in a rat model of heart failure with preserved systemic iron status but depleted intracellular cardiac stores

**DOI:** 10.1038/s41598-018-33277-2

**Published:** 2018-10-25

**Authors:** Aleksandra Paterek, Marta Kępska, Barbara Sochanowicz, Ewelina Chajduk, Joanna Kołodziejczyk, Halina Polkowska-Motrenko, Marcin Kruszewski, Przemysław Leszek, Urszula Mackiewicz, Michał Mączewski

**Affiliations:** 1 Department of Clinical Physiology, Centre of Postgraduate Medical Education, Warsaw, Poland; 2 Centre for Radiobiology and Biological Dosimetry, Institute of Nuclear Chemistry and Technology, Warsaw, Poland; 3 Laboratory of Nuclear Analytical Methods, Institute of Nuclear Chemistry and Technology, Warsaw, Poland; 4 Department of Molecular Biology and Translational Research, Institute of Rural Health, Lublin, Poland; 5 Department of Medical Biology and Translational Research, Faculty of Medicine, University of Information Technology and Management, Rzeszów, Poland; 6 Heart Failure and Transplantology Department, Institute of Cardiology, Warsaw, Poland

## Abstract

Iron deficiency (ID) commonly occurs in chronic heart failure (HF) and is associated with poor prognosis. Neither its causes nor pathophysiological significance are clearly understood. We aimed to assess iron status and the effect of iron supplementation in the rat model of post-myocardial infarction (MI) HF. Four weeks after induction of MI to induce HF or sham surgery, rats received intravenous iron (ferric carboxymaltose) or saline, 4 doses in 1-week intervals. HF alone did not cause anemia, systemic or myocardial ID, but reduced myocardial ferritin, suggesting depleted cardiomyocyte iron stores. Iron therapy increased serum Fe, ferritin and transferrin saturation as well as cardiac and hepatic iron content in HF rats, but did not increase myocardial ferritin. This was accompanied by: (1) better preservation of left ventricular (LV) ejection fraction and smaller LV dilation, (2) preservation of function of Ca^2+^ handling proteins in LV cardiomyocytes and (3) reduced level of inflammatory marker, CRP. Furthermore, iron supplementation did not potentiate oxidative stress or have toxic effects on cardiomyocyte function, but increased activity of antioxidant defenses (cardiac superoxide dismutase). Despite lack of systemic or myocardial ID we found evidence of depleted cardiomyocyte iron stores in the rat model of HF. Furthermore we observed positive effect of iron supplementation and confirmed safety of iron supplementation in this setting.

## Introduction

Iron is a vital element for the body, especially for metabolically active tissues such as myocardium. It is a crucial component of oxygen carrying protein, hemoglobin and of multiple oxidative enzymes and respiratory chain proteins, including those containing Fe-S clusters, involved in cellular metabolism. Dietary iron is absorbed by enterocytes and then secreted into circulation where it is bound to an iron transporting protein, transferrin, which on one hand delivers iron to target cells (by binding to the transferrin receptor-1 [TfR1]), on the other neutralizes its free radical generating activity. Iron can be utilized by target cells or stored, bound to ferritin, mainly in the liver. Thus transferrin saturation with iron is a good indicator of usable iron pool, while ferritin is a good indicator of total body iron (however, being an acute phase protein, it can be increased in inflammatory states).

Iron deficiency (ID), occurs in up to 50% of patients with chronic heart failure (HF), both with co-morbid anemia and with normal hemoglobin values^1^. Its etiology is likely multifactorial and remains largely unknown. Broadly speaking, ID can be attributed to the factors related to HF per se (e.g. malabsorption due to venous congestion, impaired nutrition, reduced intracellular uptake of iron due to reduced cellular TfR1, iron sequestration associated with chronic inflammation accompanying HF, and intracellular abnormalities of iron handing in cardiomyocytes) or to the factors related to co-morbidities and concomitant medications (e.g. gastrointestinal or genitourinary blood loss related to the use of antiplatelet drugs and/or oral anticoagulants)^2^. Furthermore, decreased myocardial iron content can occur despite normal parameters of systemic iron turnover. We have previously shown that myocardial iron content in HF patients is lower than expected based on systemic iron turnover parameters (ferritin concentration, transferrin saturation)^3^. To make this even more complex, there can be iron shifts between specific intracellular compartments despite normal myocardial iron content^4^.

In HF patients ID is associated with poor prognosis, independently of hemoglobin concentration^1,5,6^. Furthermore it is associated with disease symptoms, limited exercise tolerance, poor quality of life and increased risk of hospitalization^1,5^. Whereas intravenous iron supplementation has beneficial symptomatic effects, e.g. improves exercise capacity (longer distance in the 6-minute walk test), NYHA class and quality of life, its effect on hard endpoints, such as survival and left ventricular (LV) remodeling parameters, fundamental for prognosis of HF patients, is still unknown^7^.

On the other hand, iron is a highly toxic and tightly controlled element. Iron overload cardiomyopathy is a known complication of iron excess^8^ and the heart is most susceptible to iron overload. Furthermore iron can participate in the Fenton reaction to form a very reactive hydroxyl radical, so its level in the human body must be strictly controlled. Thus, the postulate that instead of iron supplementation, iron chelation should be recommended for heart diseases, was risen recently^9^. Therefore safety of treatment with intravenous iron in HF is of utmost importance.

Hence there are two unresolved issues that we aimed to address in our study. First, we wanted to characterize in detail iron status, at the cardiac, serum and hepatic level in the rat model of post-myocardial infarction (MI) HF that offered unique ability to dissect HF-specific changes of iron status from those related to other factors (such as comorbidities or concomitant medications) which was impossible in human studies. Second, we wanted to evaluate efficacy and safety of intravenous iron supplementation in HF and thus we assessed effects of 4-week intravenous iron administration on HF progression evaluated by LV remodeling, changes in hemodynamic parameters, calcium handling and cardiomyocyte contractility as well as on the biochemical parameters of iron turnover, oxidative stress and inflammation.

## Results

### Infract size, morphological parameters and mortality

**Figure 1:**
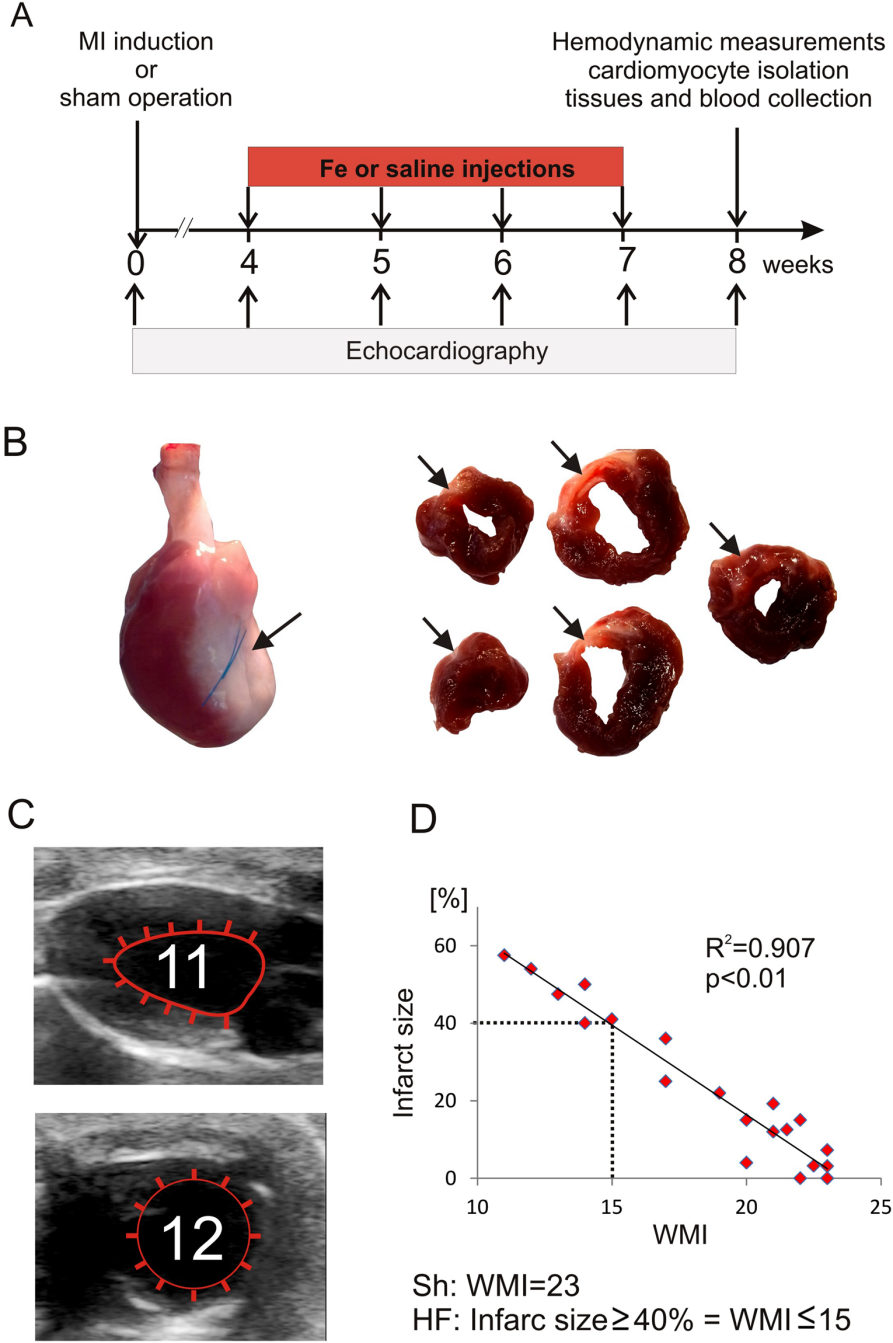
Experimental protocol and the myocardial infarction size evaluation by echocardiography. (**A**) Schematic representation of experimental protocol. The time of surgery, Fe or saline injections and echocardiographic examinations were indicated. (**B**) A representative heart 4 weeks after induction of myocardial infarction with visible collagen scar in LV free wall and in LV cross-sections (arrows); (**C**) the echocardiographic LV visualization in the long- (upper panel) and midpapillary short- (lower panel) axis view. Regional LV wall motion abnormalities were quantitated separately for 23 LV wall segments (11 and 12 segments visualized in the long- and short-axis view, respectively) and were graded as 1 point (normal) or 0 points (abnormal). The total WMI was calculated as a sum of the points. (**D**) WMI correlation with infarct size expressed as a percentage (calculated by dividing the sum of infarct areas from all sections by the sum of LV areas from all sections, including those with infarct scar, and multiplying by 100). WMI = 15 corresponded to infarct size ~40%.

Rats were subjected to MI induction (HF group) or sham operation (Sh). After 4 weeks of follow-up both Sh and HF rats were randomized into two subgroups, receiving ferric carboxymaltose (Fe) or saline (Fig. 1A, see also Method section). The mean infarct size evaluated by transthoracic echocardiography (Fig. 1B–D) in both HF groups did not differ at randomization and at the end of the study (4 and 8 weeks after MI induction, respectively) (Table 1). There were no differences in body weight between HF and Sh animals at any time point after the surgery. In HF rats pulmonary congestion, reflected by increased corrected lung weight and LV hypertrophy, as indicated by increased corrected LV weight, were found (Table 1). No death occurred in either study group after beginning of iron/saline injections.

**Table 1:** The effect of iron supplementation on the morphometric and hemodynamic parameters in failing (HF) and control rats (Sh).

	Sh (n = 15)	Sh + Fe (n = 15)	HF (n = 15)	HF + Fe (n = 15)
Infarct size at 4 week (%)	—	—	50.31 ± 0.57	50.36 ± 0.37
Infarct size at 8 week (%)	—	—	50.36 ± 0.29	50.42 ± 0.38
BW (g)	397 ± 7	398 ± 7	412 ± 10	405 ± 8
Heart/BW (mg/g)	2.63 ± 0.08	2.71 ± 0.08	3.52 ± 0.08^#^	3.45 ± 0.22^$^
Lung/BW (mg/g)	4.83 ± 0.2	4.81 ± 0.24	8.35 ± 0.83^#^	7.78 ± 0.72^$^
HR (bpm)	435 ± 31	432 ± 14	430 ± 32	426 ± 31
+dP/dt_max_ (mmHg/s)	5002 ± 265	4631 ± 251	3270 ± 167^#^	3349 ± 402^$^
−dP/dt_max_ (mmHg/s)	4195 ± 256	4278 ± 207	2708 ± 84^#^	2754 ± 243^$^
LVESP (mmHg)	105 ± 5	99 ± 3	95 ± 3	93 ± 5
LVEDP (mmHg)	5 ± 1	3 ± 1	20 ± 3^#^	18 ± 1^$^

Sh, sham operation; HF, heart failure; Fe, ferric carboxymaltose; BW, body weight; HR, heart rate; +dP/dt_max_, peak rate of left ventricular pressure increase; −dP/dt_max_, peak rate of left ventricular pressure decay; LVESP, left ventricular end-systolic pressure; LVEDP, left ventricular end-diastolic pressure. Data is represented as mean ± SEM; ^#^p < 0.05 vs. Sham, ^$^p < 0.05 vs^.^ Sham + Fe.

### Hemodynamic function and left ventricular dimensions

**Figure 2:**
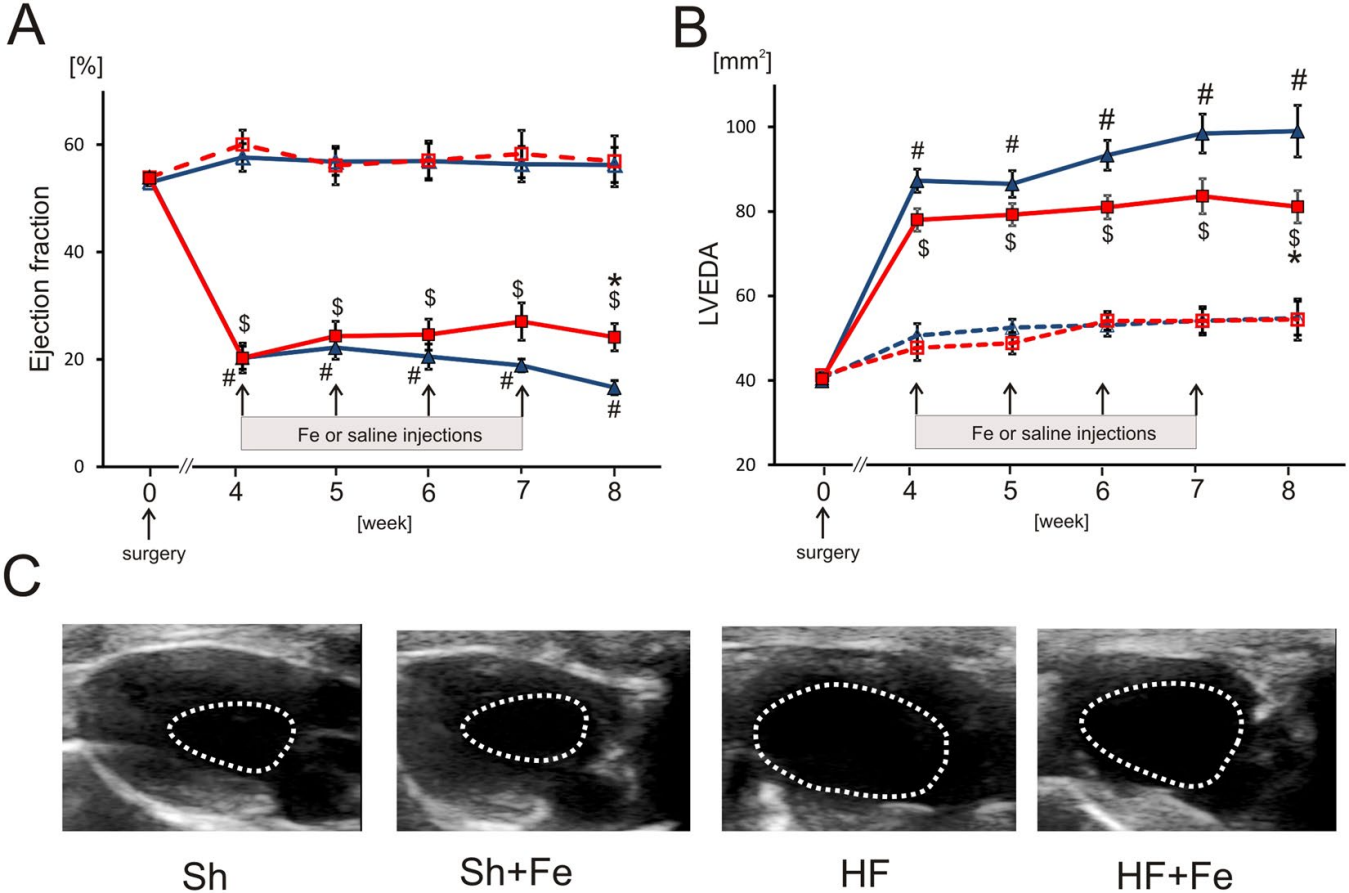
The effect of intravenous iron (Fe) supplementation on echocardiographic parameters in sham-operated (Sh) and heart failure (HF) rats. (**A**) Ejection fraction calculated as (LV end-diastolic area - LV end-systolic area)/LV end-diastolic area planimetered from the long-axis view. (**B**) left ventricular end-diastolic area (LVEDA); (**C**) the representative echocardiographic images of LV end-diastolic area from the long-axis view. Data is represented as mean ± SEM, n = 10 rats in each group, ^#^p < 0.05 vs. Sham, ^$^p < 0.05 vs. Sham + Fe, *p < 0.05 vs. HF.

Eight weeks after surgery heart rate (HR) did not differ between all Sh and HF animals (Table 1). In HF groups we observed (i) LV systolic dysfunction, as evidenced by reduced LV ejection fraction (LVEF) (Fig. 2A), contractility (+dP/dt_max_), as well as an increase of LV end-diastolic pressure (LVEDP) (Table 1); (ii) LV diastolic dysfunction indicated by reduced rate of LV pressure decay (−dP/dt_max_) (Table 1) and (iii) LV dilatation, reflected by increased LV diastolic area (Fig. 2B,C). Iron administration had no effect on hemodynamic parameters, however partially prevented deterioration of LV systolic function in the HF group, as evidenced by significantly higher LVEF (HF + Fe: 24.1 ± 7.9% vs. HF: 14.7 ± 4.1%) (Fig. 2A) and prevented progression of LV dilatation, since LV diastolic area was significantly lower (HF + Fe: 81 ± 12 mm^2^ vs. HF: 99 ± 19 mm^2^) 8 weeks after MI induction (Fig. 2B,C).

### Hematology parameters, serum iron status, inflammation marker

There were no differences between groups in hematologic parameters (Table 2). HF had no effect on systemic iron turnover, although there was a trend towards lower transferring saturation (TSAT) and serum iron concentration and significantly lower sTfR concentrations than in Sh group (Table 3).

**Table 2:** The effect of iron supplementation on the hematology parameters in failing (HF) and control rats (Sh).

	Sh (n = 8)	Sh + Fe (n = 10)	HF (n = 9)	HF + Fe (n = 10)
Hgb (g/dl)	14.6 ± 0.3	14 ± 0.4	14.6 ± 0.3	14.6 ± 0.5
Hct (%)	43.2 ± 0.9	42.3 ± 0.9	44.4 ± 0.8	43.8 ± 1.1
MCV (fl)	50.2 ± 0.3	53.3 ± 1.1	51.7 ± 0.7	52.7 ± 0.8
MCH (pg)	16.9 ± 0.1	17.4 ± 0.2	17 ± 0.2	17.6 ± 0.2
MCHC (g/dl)	33.7 ± 0.2	33 ± 0.3	32.9 ± 0.2	33.3 ± 0.3
RBC (10^6^/µl)	8.6 ± 0.2	8 ± 0.3	8.6 ± 0.2	8.3 ± 0.3
WBC (10^3^/µl)	1.3 ± 0.2	1.7 ± 0.4	1.7 ± 0.4	1.6 ± 0.3
PLT (10^3^/µl)	847 ± 103.7	1053 ± 44.5	772 ± 85.3	897 ± 39.5
Ret (%)	3.1 ± 0.2	3.8 ± 0.3	3.1 ± 0.2	3.2 ± 0.2

Hgb, hemoglobin concentration; Hct, hematocrit; MCV, mean corpuscular volume; MCH, mean corpuscular hemoglobin; MCHC, mean corpuscular hemoglobin concentration; RBC, red blood cell count; WBC, white blood cell count; PLT, platelets; Ret, reticulocyte count. Data is represented as mean ± SEM.

**Table 3:** The effect of iron supplementation on the serum parameters of iron turnover in failing (HF) and control rats (Sh).

	Sh	Sh + Fe	HF	HF + Fe
Fe (µg/dl) (n = 14)	194 ± 8	228 ± 17	178 ± 13	277 ± 15*
Ferritin (µg/ml) (n = 14)	2.17 ± 0.21	4.96 ± 0.45^#^	2.37 ± 0.35	6.75 ± 0.86*
TIBC (µg/dl) (n = 6)	448 ± 20	478 ± 18	486 ± 30	464 ± 9
TSAT (%) (n = 6)	39.5 ± 0.7	44.1 ± 4.2	35.9 ± 2.3	59.1 ± 4.2*
UIBC (µg/dl) (n = 6)	271 ± 13	265 ± 15	308 ± 9	191 ± 22*^,$^
sTfR (nmol/l) (n = 10)	4.74 ± 0.38	4.03 ± 0.28	2.36 ± 0.4^#^	2.64 ± 0.66^$^
CRP (mg/ml) (n = 10)	0.87 ± 0.04	0.94 ± 0.07	1.24 ± 0.05^#^	0.96 ± 0.04*

Fe, iron concentration; TIBC, total iron binding capacity, TSAT, transferrin saturation; UIBC, unsaturated iron binding capacity; sTfR, soluble transferrin receptor; CRP, C reactive protein. Data is represented as mean ± SEM; ^#^p < 0.05 vs. Sham, ^$^p < 0.05 vs. Sham + Fe, *p < 0.05 vs. HF.

As expected, administration of Fe resulted in increase of ferritin level in Sh and HF groups (Table 3), higher in HF than in Sh animals. In addition, in HF group Fe injections resulted in an increase of TSAT and serum iron concentration and a decrease of unsaturated iron binding capacity (UIBC).

Concentration of C-reactive protein (CRP), a marker of inflammation, was increased in HF group as compared to Sh group, while Fe prevented this increase.

### Cardiac and hepatic tissue iron content

**Figure 3:**
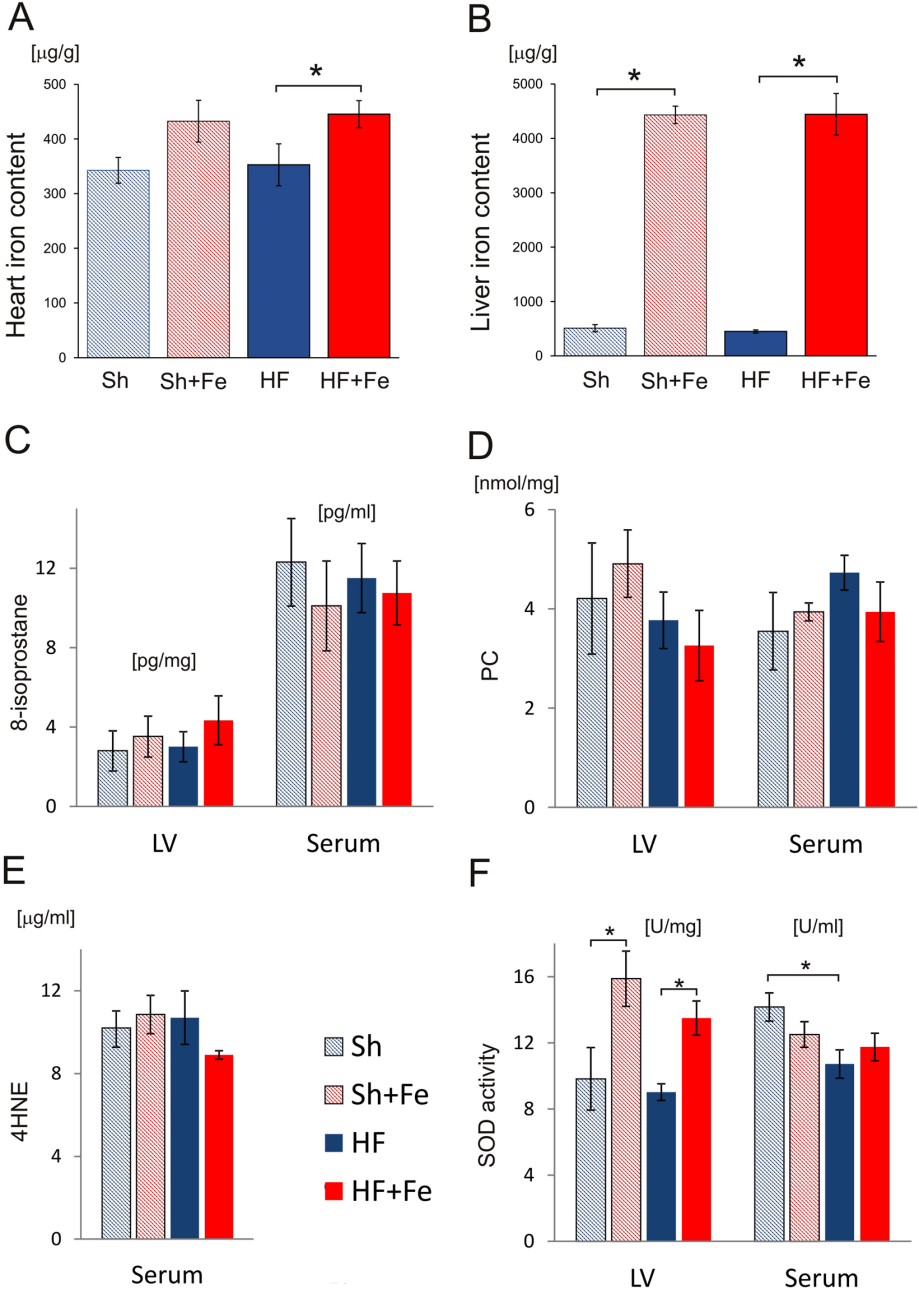
The effect of intravenous iron (Fe) supplementation on tissues Fe content and oxidative status in sham-operated (Sh) and heart failure (HF) rats. (**A**,**B**) Fe content in the myocardium and liver, respectively. Oxidative status was evaluated in the left ventricular tissue (LV) and serum: concentration of an oxidative stress marker, 8-isoprostane (**C**) oxidative protein damage by the assessment of protein carbonyl (PC) (**D**) concentration of a marker of lipid peroxidation, 4-HNE (**E**) antioxidant enzymatic activity of total superoxide dismutase (SOD) (**F**). Data is represented as mean ± SEM, the tissues were collected from n = 8–9 hearts (**A**) and from n = 5–6 livers (**B**) in each experimental group. Parameters of oxidative stress (**C**–**F**) in tissue and serum were measured in at least 5 rats in each group, *p < 0.05, only statistically significant differences were shown.

There was no change of LV myocardial or liver iron content in HF versus sham rats (Fig. 3). Fe supplementation resulted in increase of myocardial iron content only in HF group (HF + Fe: 445 ± 25 µg/g vs. HF: 326 ± 38 µg/g) (Fig. 3A), while liver iron content increased in both Fe-treated groups (Fig. 3B), by almost 10-fold in HF animals (HF + Fe: 4443 ± 383 µg/g vs. HF: 449 ± 29 µg/g) and by 8.7-fold in Sh ones (Sh + Fe: 4431 ± 161 µg/g vs. Sh: 508 ± 67 µg/g).

### Oxidative stress

To evaluate the effect of HF and iron supplementation on oxidative status, we measured in serum and LV tissue: (i) concentration of an oxidative stress marker, 8-isoprostane, formed involving free radical initiated peroxidation of arachidonic acid, (ii) oxidative protein damage by the assessment of protein carbonyl (PC) as well as (iii) antioxidant enzymatic activity of total superoxide dismutase (SOD). Additionally, (iv) concentration of a marker of lipid peroxidation, 4-HNE was measured in serum.

No difference was found in either of oxidative stress parameters in serum or LV tissue between HF versus Sh groups or saline versus Fe receiving animals (Fig. 3C–E). However, serum SOD activity in HF group was lower than in Sh group (Fig. 3F). Fe administration in both groups (Sh and HF) resulted in higher SOD tissue activity, while SOD serum activity did not change (Fig. 3F).

### Expression of iron handling proteins in cardiac tissue

**Figure 4:**
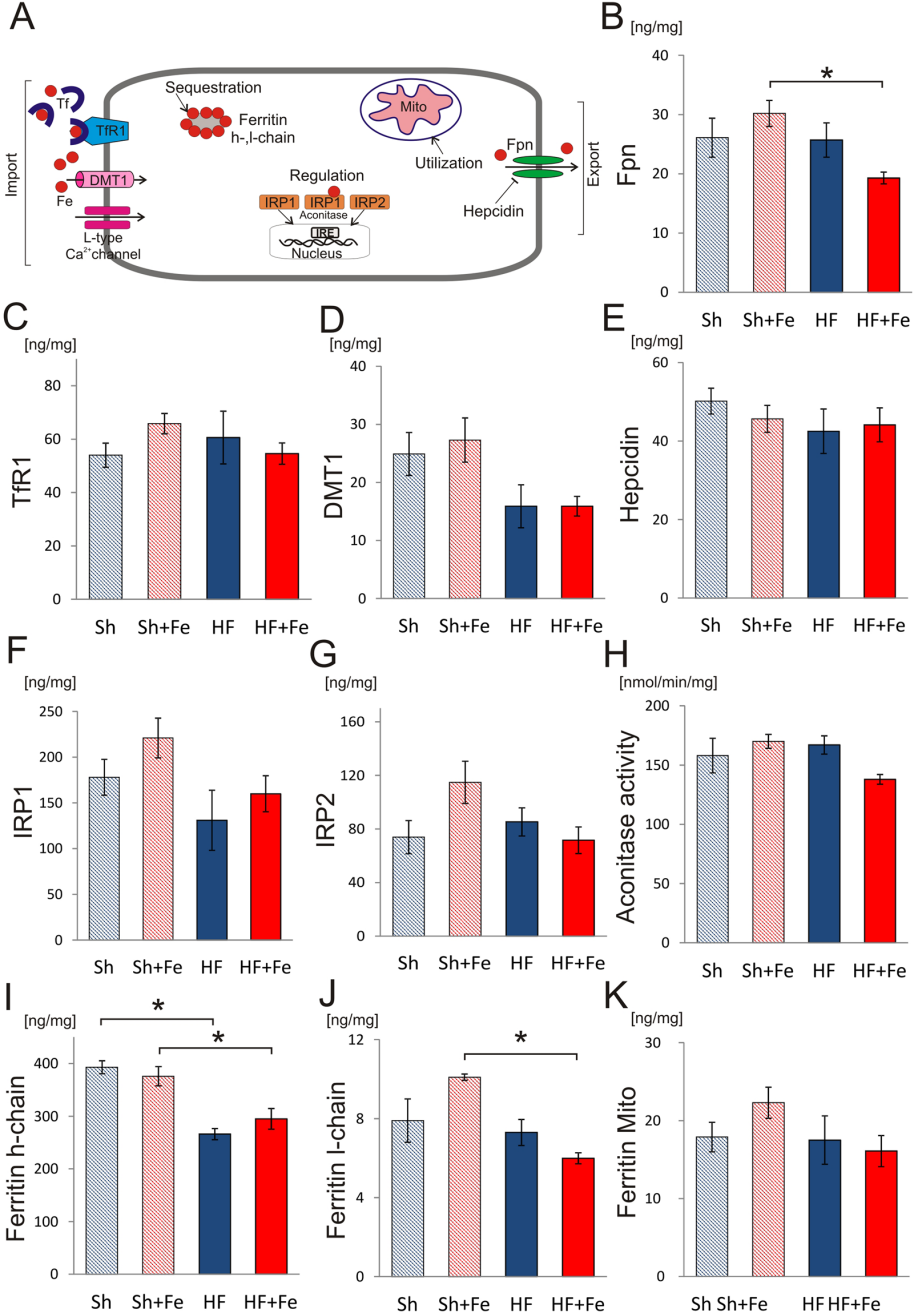
The effect of intravenous iron (Fe) supplementation on expression of the iron handling proteins in LV cardiac tissue from sham operated (Sh) and heart failure (HF) rats. (**A**) The schematic illustration of iron handling in cardiomyocytes. (**B**–**G**) Expression of ferroportin (Fpn), transferrin receptor 1 (TfR1), divalent metal transporter 1 (DMT1), hepcidin, iron regulatory protein 1 (IRP1) and iron regulatory protein 2 (IRP2), respectively. (**H**) Activity of aconitase - iron regulatory protein 1 (IRP1) containing [4Fe-4S] cluster. Expression of ferritin h-chain (**I**), l-chain (**J**) and mitochondrial ferritin (**K**). Data is represented as mean ± SEM, n = 5–6 rats in each group, *p < 0.05, only statistically significant differences were shown.

The location and functional role of proteins involved in iron turnover in cardiomyocytes were schematically presented in Fig. 4A. Ferroportin (Fpn) (iron export protein), transferrin receptor 1 (TfR1) and divalent metal transporter 1 (DMT1) (iron import proteins) were preserved in failing hearts (Fig. 4B–D). Furthermore, hepcidin and the iron regulatory proteins 1 and 2 (IRP1 and IRP2) as well as aconitase activity (that reflects amount of IRP1 protein containing 4S-4Fe cluster and thus deprived of IRE binding capacity), jointly responsible for the regulation of expression of iron handling proteins, were unchanged in HF rats (Fig. 4E–H). Only ferritin h-chain, but not l-chain (iron sequestration proteins) were decreased in HF rats as compared to Sh animals (Fig. 4I,J), while mitochondrial ferritin was unchanged in HF vs. Sh rats (Fig. 4K).

Fe supplementation had no effect on any of the tested proteins in either Sh or HF rats (Fig. 4B–K). However, Fpn, ferritin h- and l-chain expression was reduced in HF + Fe as compared to Sh + Fe animals (Fig. 4B,I and J, respectively).

These results suggest that cytoplasmic myocardial iron stores were reduced in HF (as indicated by decreased expression of major intracellular iron storage protein, ferritin h-chain), but otherwise cardiac iron turnover seemed normal. Intravenous iron supplementation had no significant effect on expression and function of major cellular iron handling proteins in the heart tissue.

### Ca^2+^ handling

**Figure 5:**
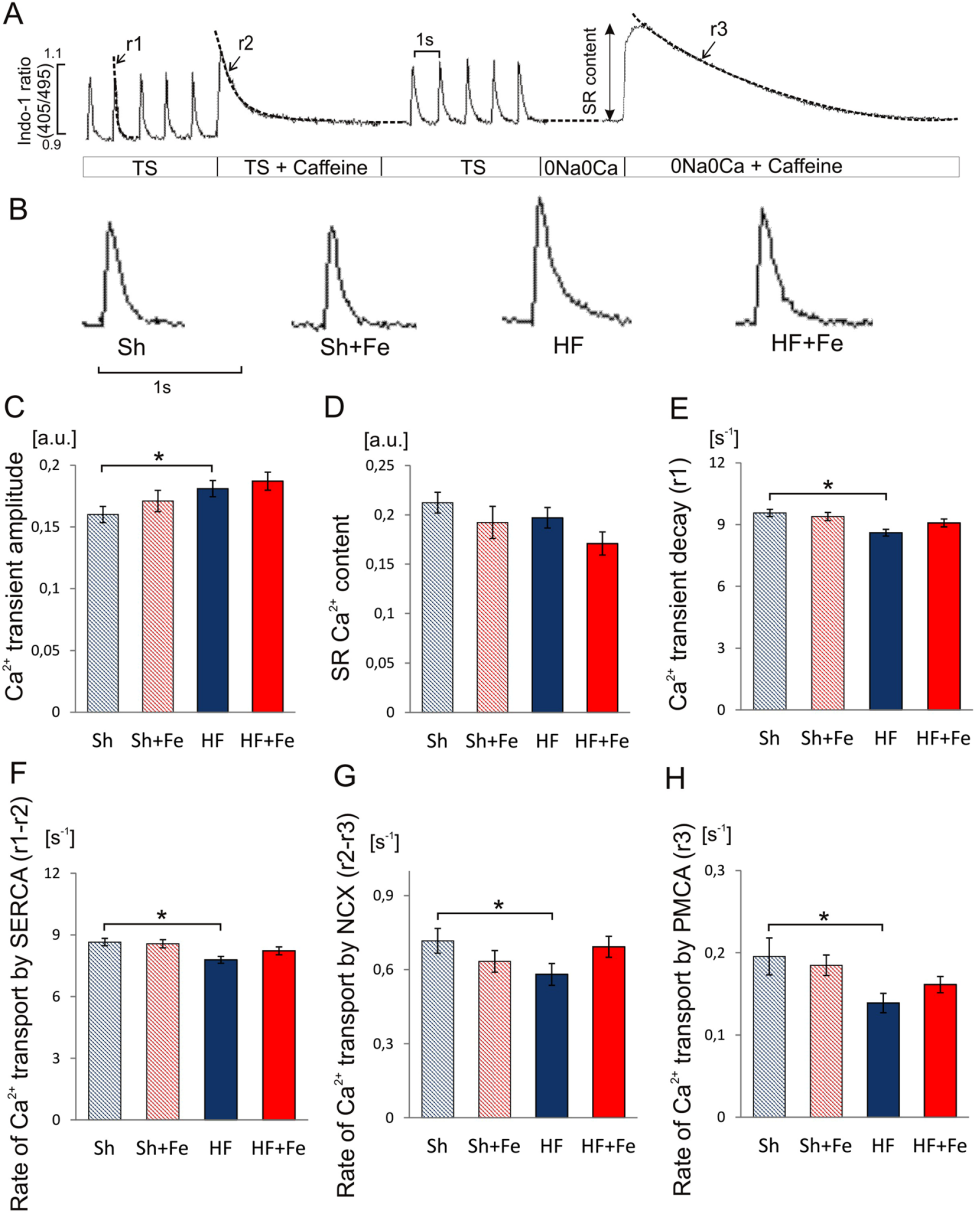
The effect of intravenous iron (Fe) supplementation on the Ca^2+^ handling parameters in cardiomyocytes isolated from sham operated (Sh) and heart failure (HF) rats. (**A**) Experimental protocol: cardiomyocytes were stimulated at 1 Hz. Caffeine was applied to cardiomyocytes superfused with Tyrode solution (TS) or Na^+^/Ca^2+^ free solution (0Na0Ca). Single exponential curves were fitted to decaying part of electrically- or caffeine-evoked Ca^2+^ transients and rate constants of their decay (r1, r2 and r3) were calculated. The rate of Ca^2+^ transport by SERCA, NCX and PMCA was calculated according to formulas: r_SERCA_ = r1 − r2, r_NCX_ = r2 − r3 and r_PMCA_ = r3, respectively. (**B**) representative Ca^2+^ transients recording for each experimental group; (**C**) amplitude of Ca^2+^ transient; (**D**) sarcoplasmic reticulum (SR) Ca^2+^ content; (**E**) rate of Ca^2+^ transient decay (r1); (**F**–**H**) rate of Ca^2+^ transport by SERCA (r1 − r2), NCX (r2 − r3) and PMCA (r3), respectively. Data is represented as mean ± SEM, n = 12–31 cardiomyocytes/group isolated from 5 hearts in each group, *p < 0.05, only statistically significant differences were shown.

To estimate the effect of iron supplementation on intracellular Ca^2+^ handling we used the protocol presented in Fig. 5A (see also Methods section). Representative Ca^2+^ transient recording in each experimental group were shown in Fig. 5B. In cardiomyocytes isolated from HF rats, the amplitude of electrically evoked Ca^2+^ transient was slightly increased as compared to the Sh rats (Fig. 5C) while a trend towards decreased SR Ca^2+^ content was observed (Fig. 5D). In HF hearts, a trend towards decreased SR content and increased amplitude of Ca^2+^ transient suggests increased fractional release, probably due to increased Ca^2+^ sensitivity of SR Ca^2+^ channels (RyRs), a phenomenon often observed in the heart failure resulting from hyperphosphorylation of RyRs.

The rate of Ca^2+^ transient decay was reduced (Fig. 5E), likely as a result of decreased rate of Ca^2+^ transport to the SR by SERCA (Fig. 5F) and diminished rate of sarcolemmal Ca^2+^ efflux through NCX (Fig. 5G) and PMCA (Fig. 5H). Fe supplementation prevented abnormalities of Ca^2+^ handling in HF rats (Fig. 5C–H). These results show that iron supplementation protects the function of Ca^2+^ transporting proteins after MI.

### Cell shortening

**Figure 6:**
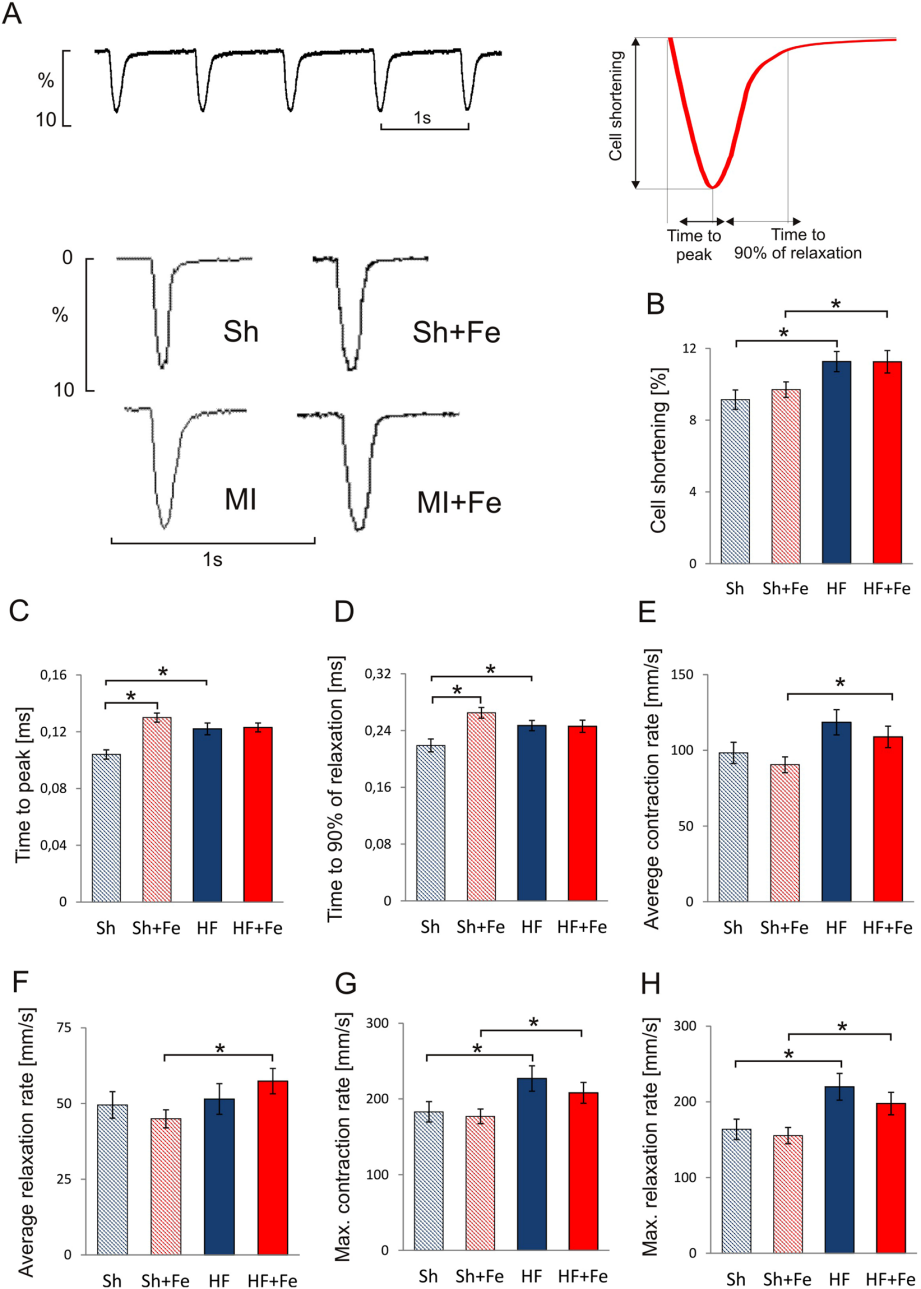
The effect of intravenous iron (Fe) supplementation on the contractile function of cardiomyocytes isolated from sham operated (Sh) and heart failure (HF) rats. (**A**) Representative recordings of cell shortening; (**B**) amplitude of cell shortening; (**C**) time to peak contraction; (**D**) time to 90% of relaxation; average contraction (**E**) and relaxation (**F**) rate; maximal contraction (**G**) and relaxation (**H**) rate. Data is represented as mean ± SEM, n = 10–23 cardiomyocytes/group from 5 hearts in each group, *p < 0.05, only statistically significant differences were shown.

Representative recordings of cell shortening in cardiomyocytes isolated from Sh and HF rats are presented in Fig. 6A. In HF cardiomyocytes the amplitude of cell shortening was increased compared to Sh myocytes (Fig. 6B). It was accompanied by prolonged contraction (Fig. 6C) and relaxation times (Fig. 6D). These results show that increased amplitude of cell shortening occurs at the expanse of longer contraction-relaxation cycle. It results in shortening of diastolic phase, which may disturb ventricular filing especially at higher heart rates.

Slower time-course of contraction-relaxation cycle could result from lower rate of contraction and relaxation due to disturbances of Ca^2+^ handling or inadequate ATP supply or from higher amplitude of cell shortening *per se*. To resolve this issue we investigated contraction and relaxation kinetics calculating average and maximal rates of contraction and relaxation phase. We found that average rates of contraction and relaxation were unchanged (Fig. 6E,F, respectively), while maximal rates were even increased (Fig. 6G,H, respectively). Thus, kinetics of contraction and relaxation in failing rat cardiomyocytes was preserved or even increased. However, this increase was not sufficient to compensate for re-lengthening of contraction and relaxation phases due to increased amplitude of cell shortening.

Fe supplementation did not influence cell shortening amplitude or time-course of either contraction or relaxation processes in HF rats (Fig. 6B–D). Additionally both average and maximal rates of contraction and relaxation were not influenced, (Fig. 6E–H, respectively).

On the other hand, in Sh rats the time to peak and the time to relaxation were longer in iron supplemented than in untreated rats (Fig. 6C,D). It was not resulted from the increased amplitude of cell shortening (Fig. 6B) and may suggest decreased rates of contraction and relaxation processes under Fe supplementation in Sh rats. Indeed, the both average and maximal rates tended to be lower in Sh +Fe than in Sh untreated rats, however the changes were statistically insignificant (Fig. 6E–H).

## Discussion

**Figure 7:**
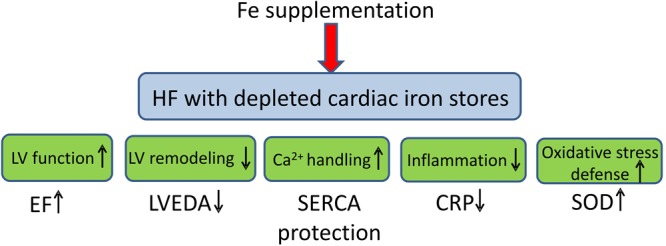
The beneficial effects of intravenous iron (Fe) supplementation in heart failure with normal iron status but depleted cardiac iron stories. Ejection fraction (EF), left ventricular end-diastolic area (LVEDA), sarcoplasmic reticulum Ca^2+^ ATP-ase (SERCA), C reactive protein (CRP), superoxide dismutase (SOD).

Here we show that:

1. In the HF model in the rat we find reduced myocardial ferritin, suggesting decreased cardiomyocyte iron stores, despite normal hematology parameters, normal systemic iron status and normal myocardial iron content.
2. Intravenous iron administration in HF provides some beneficial effects: partially prevents deterioration of EF and LV dilation, prevents impairment of Ca^2+^ handling proteins in the LV cardiomyocytes and reduces level of inflammatory marker, CRP. Furthermore, it does not potentiate oxidative stress or have toxic effects on cardiomyocyte function (Fig. 7). Thus, although we do not find evidence of either anemia or ID in HF rats, we see positive effects of iron supplementation in such setting and we demonstrate that such supplementation is safe.

Up to 50% patients with chronic HF may be ID at the systemic level, though mechanisms underlying ID in chronic HF are not fully understood^5^. Furthermore, normal systemic iron stores do not guarantee adequate myocardial iron. Indeed we^3^ and others^10^ have previously shown that myocardial iron content in explanted human failing hearts may be decreased despite normal systemic iron stores. Furthermore, data concerning iron status in explanted failing hearts are not consistent. Chang *et al*.^11^ found identical cytosolic iron content in cardiac samples from patients with ischemic cardiomyopathy and healthy controls and Khechaduri *et al*.^4^ even found increased cytosolic iron content in explanted failing hearts. However, others have shown that in myocardial samples from explanted failing hearts^3,4,12,13^ myocardial iron content was reduced by up to 30%.

Thus, to gain detailed insight into iron status in HF, we utilized a commonly used rat model of post-MI HF that offered pathophysiology and characteristics similar to human HF^14^, but had the advantage of identical genetic background, lack of co-morbidities, concomitant therapy and variable diet. In this model we found that HF, characterized by low LVEF, pulmonary congestion and left ventricular dilation, was accompanied by normal hematology parameters, normal serum iron parameters (iron concentration, ferritin concentration, transferrin saturation), unchanged liver iron stores, unchanged myocardial iron content and preserved expression of most of the cardiomyocyte iron handling proteins (those involved in cellular iron regulation (IRP1, IRP2, hepcidin), import (TfR1, DMT1), export (Fpn) and sequestration (ferritin-l)). However, cardiac ferritin-h, major cellular iron storage protein, was reduced by almost 30%, indicating reduced cytoplasmic iron stores. To gain further insight into intracellular iron compartmentalization, we investigated expression of the mitochondrial ferritin, but found it unchanged. Normal systemic and cardiac iron content, but reduced cytoplasmic cellular stores may suggest displacement of cytoplasmic iron to mitochondrial compartment due to greater iron utilization in HF hearts.

Indeed, Khechaduri *et al*.^4^ revealed increased mitochondrial iron content in failing human hearts, but while mitochondrial non-heme iron was unchanged, mitochondrial heme iron was increased. This was associated with increased activity of δ-aminolevulinic acid synthase (ALAS), the rate limiting step of heme synthesis. Thus in failing hearts iron utilization may be increased and channeled into heme production at the cost of other iron-requiring processes, such as production of Fe/S clusters containing enzymes. In this context Haddad *et al*.^12^ found that reduction of myocardial iron content resulted in preferential reduction of Fe/S-cluster containing complex I activity in mouse hearts. Thus increased ALAS activity, probably due to repeated ischemia related to HF, may result in increased heme production, leading to increased utilization of iron (and hence depletion of iron stores) and reduced availability of iron for other processes and signs of functional cytoplasmic iron deficiency.

Several clinical trials demonstrated that intravenous iron supplementation successfully improved exercise tolerance, maximum oxygen consumption or functional NYHA class^15,16^ in HF patients with impaired EF and ID, though effect on hard endpoints, such as mortality, was not studied, while oral iron supplementation was ineffective^17^.

Here we offer a novel observation that intravenous iron supplementation may be effective, i.e. partially prevent deterioration of LV contractility and LV dilation, impairment of activity of Ca^2+^ handling proteins in the LV cardiomyocytes and reduce inflammation in the rat model of HF despite normal systemic iron status and normal myocardial iron content, but reduced myocardial iron stores.

There are several possible mechanisms that could explain this phenomenon. First, HF is known to be associated with mitochondrial dysfunction^18^, which could be of particular importance since HF is accompanied by increased preload and afterload, putting metabolic stress on the heart. As provided above, in failing hearts production of Fe/S clusters containing enzymes may be impaired by preferential production of heme-containing enzymes. Haddad *et al*.^12^ demonstrated that iron supplementation could possibly restore at least some of these defects. Second, reports indicate that oxidative stress is increased in HF^19^. Its possible negative consequences include detrimental modification of calcium handing proteins and overall cellular dysfunction. Dysfunctional mitochondria are a major source of oxygen free radicals in failing hearts^11^. Furthermore, oxidative stress might directly affect some Fe-S cluster containing proteins, potentiating their inhibition even more. Both iron deficiency and iron excess stimulate oxidative stress^20^. Hence oxidative stress may exacerbate mitochondrial dysfunction and be a part of a detrimental positive feedback loop. We can hypothesize that iron supplementation, through increased antioxidant defenses or improvement of mitochondrial function, could reduced this detrimental oxidative stress, although this mechanism is unlikely to contribute to beneficial effects of iron supplementation in our model since we did not find evidence of increased oxidative stress, however increased SOD activity both in Sh and HF rats receiving iron in our model is intriguing here.

Mitochondrial ferritin is especially interesting in this context. This protein acts probably not as an iron storing protein, but as a scavenger of labile iron to reduce the risk of free radical production. It is especially abundant in mitochondria with high respiratory activity and its expression rises rapidly with increased ATP production and oxidative stress^21^. Expression of mitochondrial ferritin did not change in our failing hearts, supporting lack of increased oxidative stress. Furthermore iron supplementation did not result in its overexpression, thus suggesting that iron therapy did not provoke increased oxidative stress.

Third, HF is associated with mild inflammation^22^. Indeed, we observed increased serum C-reactive protein concentration in our model. Iron supplementation could attenuate inflammation through action on immune cells. Weiss *et al*.^23^ demonstrated *in vitro* that iron availability influenced immune effector functions: in an iron-deprived situation, macrophages responded stronger to stimulation with Th1-type cytokine interferon-gamma, i.e. pathways driven by interferon-gamma were super-induced in iron-deficiency states. Vice versa, when iron was supplemented, pro-inflammatory cascades were dampened. In this context interferon gamma overexpression was shown to induce abnormalities of Ca^2+^ handling and contractility^24^ and hence reduction of inflammation mediated by increased iron availability could be at least partially responsible for our results.

In our study we provided the same per kg dose of intravenous Fe, as was used in human HF clinical trials^15,16^. It was effective, i.e. successfully increased body iron stores, which was indicated by increase of hepatic iron content by almost 10-fold both in sham operated and HF rats, but was not excessive, since it only mildly increased serum ferritin and free iron concentration; increase of myocardial iron content amounted to no more than approximately 20% in HF, while in sham operated rats failed to reach significance. Furthermore this dose of intravenous ferric carboxymaltose was safe as it did not increase mortality, oxidative stress or induce toxic effects at the cardiomyocyte level. Moreover, up to 60-fold increases of hepatic iron content appeared to be non-toxic in other studies^25^.

Safety of iron supplementation is of major importance in the context of cardiomyocyte contractile function. Cardiomyocytes are especially prone to iron overload. In most cells iron uptake is achieved mainly by internalization of transferrin receptors upon binding of transferrin-iron complex. This uptake is tightly regulated, i.e. elevation of serum iron results in reduction of transferrin receptor expression. This self-limiting mechanism protects cells against iron overload. However, additional non-transferrin bound iron transport, such as DMT1 and ion channels able to permit divalent metal cations exist in many cells. While DMT1 is weekly expressed in cardiomyocytes and similarly to transferrin receptors, negatively regulated by increased serum iron level, L-type Ca^2+^ channels are permeable for Fe^2+^ cations and are widely expressed in cardiomyocyte sarcolemma. They play a major role in calcium induced calcium release, crucial for cardiomyocyte contraction. Moreover, the entry of iron through L-type Ca^2+^ channels is an element of positive feedback^26^. Iron ions passing through Ca^2+^ channel enter the cell and inhibit the Ca^2+^-dependent inactivation of L-type Ca^2+^ current, promoting increased permeability of these channels for both Ca^2+^ and Fe^2+^ ^27^. Thus elevated non-transferrin bound serum iron level may lead to cardiomyocyte iron overload accompanied by Ca^2+^ overload, both impairing cardiomyocyte Ca^2+^ handling and contractile function. Furthermore, Ca^2+^ handling proteins, namely SERCA2a and RyRs, are especially prone to oxidative post-translational modifications, which could be of importance in the context of iron overload related increase of oxidative stress.

According to Li *et al*.^28^ the first sign of myocardial iron overload is diastolic dysfunction due to reduction of SERCA activity and impairment cardiomyocyte relaxation. Also Wongjaikam and colleagues^29^ have shown that level of SERCA protein in the heart was significantly reduced in iron-overloaded rats. Thus, cardiomyocyte function and Ca^2+^ handling are sensitive sensors of iron excess in cardiomyocytes. In this context, we did not find any signs of iron overload in iron supplemented rats. Actually, quite opposite was true: Ca^2+^ transport by SERCA and the rate of Ca^2+^ transient decay were preserved in HF + Fe rats compared to HF rats and there was no Ca^2+^ overload in iron-supplemented groups.

Surprisingly, iron supplementation affected more noticeably cardiomyocyte contractile performance in Sh than HF rats, prolonging both contraction and relaxation time. It might suggest that iron supplementation in the setting of normal iron status is even safer in respect to cardiomyocyte contractile function in failing than healthy animals.

Despite lack of any signs of iron toxicity in our HF model, results of iron supplementation in HF rats indicate lower “systemic iron buffering capacity” in this setting, i.e. administration of the same dose of FCM per kg body weight resulted in a significant increase of serum iron concentration and TSAT in HF rats, while neither of these parameters was significantly increased in Sh rats. Furthermore, increase of serum ferritin concentration in HF rats was significantly higher than in sham operated rats. Moreover, increase of myocardial iron content was significant in HF rats but failed to reach significance in Sh rats. However, hepatic iron content increased to almost identical degree in both post-MI study groups and myocardial ferritin-h did not change with iron therapy, suggesting that additional iron was efficiently utilized by the cardiomyocytes. This suggests that although there are no signs of myocardial iron overload with iron therapy, systemic iron may be less efficiently channeled into tissues. Thus iron therapy needs to be carefully monitored in HF to prevent potential iron overload.

## Conclusion

Although we did not find abnormalities of either myocardial iron content or systemic iron status in the rat model of post-MI HF, we found depleted myocardial iron stores, as indicated by reduced intracellular cytoplasmic ferritin. Furthermore, we observed the positive effect of iron supplementation on LV function, dimensions, cardiomyocyte Ca^2+^ handling and inflammation (Fig. 7). Iron therapy was safe, i.e. did not potentiate oxidative stress and did not produce signs of iron toxicity. This shows that abnormalities of iron status in HF can be very complex and certainly further studies are required to find more suitable and sensitive biomarkers of the iron status in failing hearts not only at the systemic but also at the tissue, cellular and sub-cellular levels.

## Methods

Male Wistar rats (n = 120), 280–320 g, were used in this project. All animal procedures conformed to the guidelines from Directive 2010/63/EU of the European Parliament on the protection of animals used for scientific purposes. The study was approved by the local ethics committee (Second Warsaw Local Ethics Committee for Animal Experimentation). Rats were fed with a standard chow, containing 150 mg iron per kg.

### Experimental design

Rats were subjected to baseline echocardiographic imaging followed by MI induction (HF group) or sham operation (Sh). After 4 weeks of follow-up, the animals underwent subsequent echocardiographic imaging. Only rats with large MI (≥40% of the LV) were enrolled to HF group in this study (10 rats were excluded). Both Sh and HF rats were randomized into two subgroups: group one receiving 4 doses in weekly intervals of ferric carboxymaltose (Fe, Ferinject®, 10 mg/kg body weight, 0.5 ml) as a bolus injection to the femoral vein (n = 30), and group two receiving placebo (normal saline, n = 30). Echocardiography was performed weekly. Eight weeks after the surgery rats underwent final echocardiographic imaging and LV catheterization. Blood was collected, ketamine HCl/xylazine overdose was given to euthanize the animals and subsequently hearts were excised for biochemical and cellular studies. Liver was also collected for further analysis. See Fig. 1A for the study protocol.

### Induction of myocardial infarction

Rats were anaesthetized with ketamine HCl and xylazine (100 mg/kg body weight [b.wt.], ip), left thoracotomy was performed, the heart was externalized, and a suture (5–0 silk) was placed around proximal left coronary artery. In Sh animals, it was left loose, and in MI animals, it was tied^30^. Buprenorphine (1 mg/kg b.wt., ip) was given as a postoperative analgesia. Within 4 weeks post-MI mortality was 42%. The vast majority of these deaths (18 animals) occurred during first 2 days after induction of MI and only 4 rats died after 48-hour mark and before the 4th week after MI induction. No death occurred after randomization to iron/saline groups 4 weeks after MI induction and completion of the study (Fig. 1A). Four weeks after MI induction the collagen scar was formed and clearly visible in the free LV wall and in the LV cross-sections (Fig. 1B)

### Echocardiography imaging

Echocardiography was performed using MyLab25 (*Esaote*, Italy) with 13 MHz linear array transducer. Each rat was examined before as well as 4, 5, 6, 7, and 8 weeks after the surgery. Under light isoflurane anesthesia LV end-diastolic and end-systolic diameters were determined from the long-axis view at the aortic valve level. Regional LV wall motion abnormalities were assessed using the wall motion index (WMI). Contractility of 12 wall segments visualized in the midpapillary short-axis view and 11 segments visualized in the long-axis view was graded as 1 (normal) or 0 (abnormal) and the total WMI was calculated (Fig. 1C). The normal hearts had WMI = 23. The measurements conducted in this study and our previous results^31^ revealed that WMI closely correlated with infarct size and that WMI = 15 corresponded to infarct size ~40% (Fig. 1D).

LV ejection fraction (LVEF) was calculated as (LV diastolic area - LV systolic area)/LV diastolic area. All measurements were obtained by one observer blinded to the study groups.

### Hemodynamic measurements

Under light anesthesia (ketamine HCl and xylazine100 mg/kg b.wt., ip) a micromanometer-tipped catheter (SPC-320, *Millar Instruments Inc*.), was advanced through the right carotid artery into the LV for recording of LV end systolic and end diastolic pressures and peak rate of rise and decline of LV pressure (+dP/dtmax and −dP/dtmax).

### Hematology

Blood samples were drawn from the heart using a syringe with 21-gauge needle. Blood was collected into tubes with lithium heparin and analyzed using a hematological autoanalyzer (XT 2000i, *Sysmex*) to determine: hemoglobin (Hgb), hematocrit (Hct), red blood cell count (RBC), mean corpuscular volume (MCV), mean corpuscular hemoglobin (MCH), mean corpuscular hemoglobin concentration (MCHC), platelet count (PLT) white blood cell count (WBC), and reticulocyte count (Ret).

### Serum iron status

Additional blood was collected into tubes with clot activator and gel separator. Cobas 8000 c701 (*Roche*) analyzer was used to determine iron concentration and unsaturated iron-binding capacity (UIBC). Transferrin saturation (TSAT) was calculated using formula: TSAT (%) = (Serum Iron/Total Iron Binding Capacity) ×100%. Total iron binding capacity (TIBC) was calculated as the sum of serum iron and UIBC values. Concentrations of serum ferritin and soluble transferrin receptor were measured using commercially available Elisa assays (Ferritin Rat Elisa Kit, *Abnova* and sTfR Immunoassay, *R&D Systems*) according to the manufacturer’s instructions.

### Heart tissue and liver iron content

Liver and heart samples were ground to a powder in liquid nitrogen, dried in the vacuum centrifuge and placed in a nitric acid-washed tube. The concentration of iron in samples was determined using an instrumental neutron activation analysis (INAA). Samples, elemental Fe standards and certified reference materials (CRMs, Bovine Liver) were irradiated for 45 min at the thermal neutron flux 10^14^ cm^−1^ s^−1^ in Polish reactor MARIA. Two weeks after irradiation gamma-ray spectrometric measurements were performed using HPGe detector coupled to gamma-ray spectrometer (*CANBERRA*). The duration of each measurement was from 6000 to 60000 s.

### Iron handling proteins expression and activity in cardiac tissue

Heart tissue (50–100 mg) was homogenized in the buffer (10 µl/mg tissue) containing: 20 mM HEPES pH 7.9, 1.5 mM MgCl_2_,10 mM KCl, 0.5 mM EDTA, 2% glycerol, 1% NP-40 (all chemicals from *Sigma*) and Complete Protease Inhibitor Cocktail (*Thermo Scientific*). Homogenate was centrifuged 20000 × g for 20 min. Supernatant was collected, portioned and frozen in liquid nitrogen. The total protein concentration were determined by Bradford method.

Aconitase activity was measured spectrophotometrically by monitoring the disappearance of cis-aconitate at 240 nm in 50 mM HEPES pH 7.4 (*Sigma*), 0.4 mM cis-aconitate (*Sigma*) and 80 µg protein extract at room temperature. Units represent nanomoles of substrate consumed per minute (ε_240 nm_ = 3.6 mM^−1^ cm^−1^).

Iron responsive element binding protein (IRP) 1 and 2, hepcidin, transferrin receptor 1 (TfR1), divalent metal transporter 1 (DMT1), ferroportin (Fpn) as well as ferritin light (l) chain, ferritin heavy (h) chain and mitochondrial (Mito) ferritin concentrations were assayed by ELISA according to the manufacturer’s instructions (all from *Cloud-Clone*).

### Oxidative stress and inflammation

Fresh liver and LV was quick-frozen in liquid nitrogen. LV tissues sample (50 mg) was homogenized in phosphate buffer with 1 mM EDTA (*Sigma*) and 0.005% BHT (*Sigma*) with 5 mm stainless steel beads in a TissueLyser LT (*Qiagen*). The homogenate was centrifuged at 14000 × g at 4 °C for 10 min, and supernatant was collected for further analysis. Total heart protein was measured using the Pierce BCA protein assay kit (*Thermo Scientific*).

The extent of lipid peroxidation was evaluated in serum and heart tissues by measuring the concentration of 4-hydroxynonenal and total 8-isoprostanes (Rat 4 Hydroxynonenal Elisa kit, *BlueGene Biotech* & 8-Isoprostane EIA Kit, *Cayman Chemical*, respectively). Before 8-isoprostane determination, samples were hydrolyzed and afterwards purified by 8-isoprostane Affinity Sorbent (*Cayman Chemical*). The level of protein carbonyl formation in LV lysates and serum (10 µg/ml) was determined using an Oxiselect protein carbonyl ELISA kit (*Cell Biolabs*). The antioxidant enzymatic activity of total superoxide dismutase (SOD) was determined in serum and cardiac tissues using commercially available Assay Kits (*Cayman Chemical*) following the manufacturer’s instructions. One unit of SOD is defined as the amount of enzyme needed to exhibit 50% dismutation of the superoxide radical.

Serum C-reactive protein was measured using a Rat C-Reactive Protein ELISA kit (*Cloud- Clone*) according to the manufacturer’s instruction.

### Myocyte isolation, recording of Ca^2+^ transient and cell shortening, evaluation of Ca^2+^ transporters activity

The heart was excised and LV cardiomyocyte were isolated according standard procedure^9^ and incubated for 15 min with 10 mM Indo-1 acetoxymethyl ester (*Sigma*) and superfused at 37 °C with Tyrode’s solution containing 1.8 mmol/L Ca^2+^. The difference between the systolic and diastolic Indo-1 fluorescence (excited at 365 and measured as ratio of fluorescence at 405 and 495 nm) was used as a measure of the amplitude of Ca^2+^ transients.

The rate of Ca^2+^ transport by sarcoplasmic reticulum (SR) Ca^2+^ -ATPase (SERCA), Na^+^/Ca^2+^ exchanger (NCX) and plasma membrane Ca^2+^-ATPase (PMCA) was estimated from the rate constants (r1, r2, r3) of the single exponential curves fitted to electrically- and caffeine-evoked Ca^2+^ transients decay, as presented in Fig. 5A.The rate constants of the Ca^2+^ transient decay for SERCA and NCX was calculated according to formulas: r_SERCA_ = r1 − r2 and r_NCX_ = r2 − r3, respectively, while r3 was taken as the measure of the rate of Ca^2+^ transport by PMCA (r3 = r_PMCA_). r_SERCA_, r_NCX,_ and r_PMCA_ describe the average velocity of Ca^2+^ transport.

SR Ca^2+^ content was estimated from the amplitude of caffeine-evoked Ca^2+^ transients in myocytes superfused with Na^+^-, Ca^2+^-free (0Na0Ca) solution.

### Recording of myocyte contractions

The myocytes contractions elicited by electrical pacing at 1 Hz were recorded by a video-edge detection system (*IonOptix* LLC, Milton USA). Amplitude and time-course of the cell shortening were analyzed by the IonWizard software (*IonOptix*). Cell shortening was expressed as a percentage of the resting cell length. The contraction time (time-to-peak) was calculated as the time from the initiation of contraction to the maximal cell shortening. The time required for re-lengthening cell to the 90% of resting cell length was taken as the relaxation time. The maximal and average rate of contraction and relaxation were evaluated to describe kinetics of contraction and relaxation process.

### Statistical analysis

All data are expressed as means ± SEM. Normal data distribution was verified by Shapiro–Wilk test while homogeneity of variances by Bartlett’s test. Differences among groups were tested by one-way analysis of variance with a Tukey test as post-hoc test or Kruskal Wallis test with a post-hoc Dunnett-Tukey-Kramer test (R version 3.2.0). P < 0.05 was accepted as a level of significance.

## Data Availability

All data generated during this study are included in this published article. The datasets generated during the current study are available from the corresponding author on demand.
